# Metastatic Cholangiocarcinoma Presenting as Punched-out Lesions of the Skull

**DOI:** 10.4274/balkanmedj.galenos.2020.2020.4.90

**Published:** 2020-08-11

**Authors:** Hirohisa Fujikawa, Makoto Araki

**Affiliations:** 1 Department of Medical Education Studies, International Research Center for Medical Education, Graduate School of Medicine, The University of Tokyo, Tokyo, Japan; 2 Department of Internal Medicine, Suwa Central Hospital, Nagano, Japan

A 60-year-old female with diabetes presented to the department of internal medicine with a 1-month history of headache. The patient had an Eastern Cooperative Oncology Group performance score of 1. Moderate tenderness was observed by soft palpation of the skull. Laboratory investigations revealed an elevated level of alkaline phosphatase (604 U/L) which was far above normal range (100-350 U/L). The serum level of hemoglobin, creatinine and calcium were within normal range. Plain radiography and computed tomography (CT) showed many punched-out osteolytic lesions in the skull (Figure 1). Cranial magnetic resonance imaging depicted multiple skull lesions with T1 hypointense signal, T2 slightly hyperintense signal, and restricted diffusion (Figure 2). Although we suspected multiple myeloma, monoclonal protein was not detected in serum. Contrast-enhanced chest-abdomen-pelvis CT revealed multiple hepatic masses, metastatic lymph nodes, and distant metastases including vertebral bodies (Figure 3). The workup for other malignant tumor was negative. Ultrasound-guided biopsy of the hepatic tumor indicated cholangiocarcinoma. The patient underwent chemotherapy with gemcitabine, in combination with radiotherapy, but died one month after admission. The patient's husband's consent was obtained.

Punched-out lesions are osteolytic lesions without sclerotic rim on X-ray examination which are considered as hallmarks of multiple myeloma. Punched-out lesions are created by the absence of reactive bone formation as a consequence of tumor factors that combine to activate osteoclasts and inhibit osteoblasts (1). Therefore, any malignancy can cause punched-out lesions. Majority of lytic bone metastases originate from the breast, lung, kidney, colon, melanoma and thyroid. However, punched-out lesions in the skull arising from cholangiocarcinoma has not been previously reported.

Cholangiocarcinoma is a heterogeneous group of tumor derived from bile ducts cells, and represents the second most frequent malignant liver cancer. Incidence is globally increasing with a rate of 2.1/100,000 person-years in Western countries. Patients with cholangiocarcinoma are known to commonly present locally advanced diseases (2). Cholangiocarcinoma usually metastasizes to the regional lymph nodes through lymphatic vessels; followed by hematogenous metastasis to the liver, peritoneum and lungs. Distant metastasis of this tumor is uncommon. However, according to a few case reports, lytic bone metastasis can be the first clinical presentation of cholangiocarcinoma (3).

## Figures and Tables

**Figure 1:**
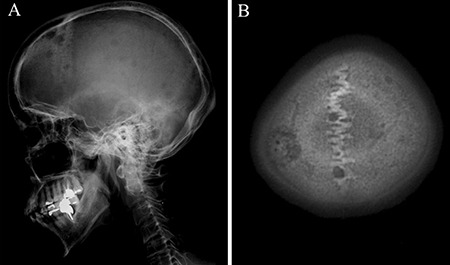
**A, B.** Lateral skull radiograph showing punched-out lesions (A), and computed tomography of the head showing multiple osteolytic lesions (B).

**Figure 2:**
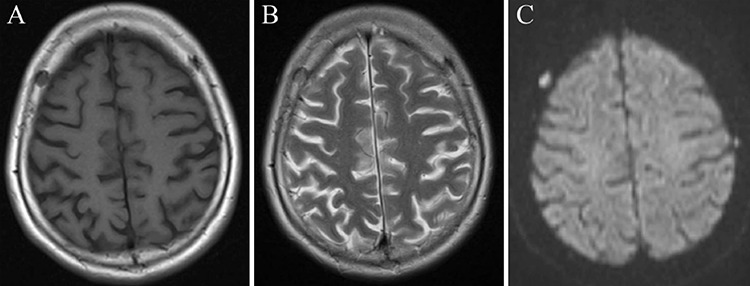
**A-C.** Cranial magnetic resonance imaging depicting multiple skull lesions with T1 hypointense signal (A), T2 slightly hyperintense signal (B), and diffusion-weighted imaging hyperintense signal (C).

**Figure 3:**
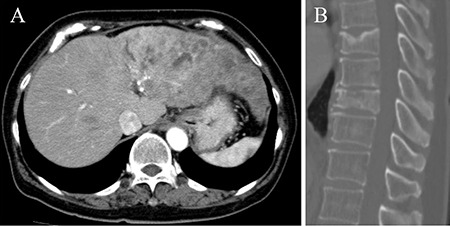
**A, B.** Axial contrast-enhanced computed tomography of the abdomen showing multiple liver masses (A), and sagittal contrast-enhanced computed tomography of the abdomen showing vertebral bodies metastases (B).
